# Symptoms of anxiety and depression among osteoporotic women

**DOI:** 10.1192/j.eurpsy.2024.1009

**Published:** 2024-08-27

**Authors:** A. Feki, I. Sellami, B. Trabelsi, Z. Gassara, S. Ben Djemaa, A. Abbes, M. Ezzeddine, M. H. Kallel, H. Fourati, R. Akrout, Y. Mejdoub, S. Baklouti

**Affiliations:** ^1^Rheumatology; ^2^Occupational medicine, Hedi Chaker Hospital; ^3^ Medecine University; ^4^Preventive medicine, Hedi Chaker Hospital, Sfax, Tunisia

## Abstract

**Introduction:**

Osteoporosis (OP) prevalence is on the rise as a result of an ageing population and lifestyle factors such as inactivity. Previous research has reported OP in individuals with depressive symptoms. Furthermore, OP has been shown to be a risk factor for anxiety.

**Objectives:**

In this study, we aimed to describe anxiety and depression symptoms among osteoporotic women in a university hospital in Tunisia.

**Methods:**

A cross-sectional study was conducted between January and June 2023 in a university hospital in Tunisia. Women with postmenopausal OP in the rheumatology department were interviewed. A hospital anxiety and depressive scale was used to describe anxiety and depression symptoms among patients. It consists of seven items for depression (HADS-D) and seven items for anxiety (HADS-A). For each component a score ≤ 7 indicated the absence of symptomatology.

**Results:**

Seventy-two women diagnosed with post-menopausal OP participated in the study. The mean age was 72.5 (±1.08). The median duration of menopause was 23 years (IIQ= [10.5-28.5]). All patients were receiving bisphosphonates. Fifty-eight women (80.5%) were identified with depressive symptoms. The median depression score was 17.5 (IIQ= [9-19]). Physical activity was significantly and inversely associated with the presence of depressive symptoms (r= -6.36; p=10^-3^). Those who were overweight or even obese had significantly more depressive symptoms than those who were not overweight (94%, 57%, p= 0.001).

The median score of anxiety was 16 (IIQ= [9-17]). Sixty-three patients (87.5%) were identified with anxiety symptoms. Physical activity was significantly and inversely associated with the presence of anxiety symptoms (r= -4.89; p=10^-3^). Women who had bone fractures were significantly more anxious than those without a history of bone fractures (100%, 63%, p<10^-3^). Patients who were overweight were significantly more anxious than those with normal weight (96%, 57%, p<10^-3^).

**Conclusions:**

Physical activity and obesity were associated with depression and anxiety among osteoporotic patients. These data are consistent with previous findings That’s why, promoting physical activity and weight loss is essential to preventing mental disorders among osteoporotic women.

**Disclosure of Interest:**

None Declared

